# Clinical characteristics and management of primary mediastinal cysts: A single‐center experience

**DOI:** 10.1111/1759-7714.13555

**Published:** 2020-07-17

**Authors:** Xun Wang, Yun Li, Kezhong Chen, Fan Yang, Jun Wang

**Affiliations:** ^1^ Department of Thoracic Surgery Peking University People's Hospital Beijing China

**Keywords:** Bronchogenic cyst, primary mediastinal cyst, thymic cyst, video‐assisted thoracic surgery (VATS)

## Abstract

**Background:**

In this study we aimed to assess the clinical outcomes of performing video‐assisted thoracic surgery (VATS) to treat primary mediastinal cysts (PMCs) and investigate the clinical factors which increase the difficulties associated with VATS.

**Methods:**

The medical records of all consecutive PMC patients, who underwent surgical resection from April 2001 to July 2016, were reviewed and 282 patients were included. Clinical characteristics, imaging features, and surgical outcomes were analyzed. Follow‐up data were successfully obtained from 230 PMC patients by telephone or outpatient clinic annually. The latest follow‐up was July 2019.

**Results:**

VATS was performed in 278 patients and four patients were converted into thoracotomy. The mean operation time and intraoperative bleeding were 102.4 ± 40.9 minutes (range 25–360 minutes) and 52.4 ± 75.1 mL (range 5–600 mL), respectively. The intra‐ and postoperative complication rates were 2.8 and 5.7%, respectively. Seven patients with bronchogenic cysts showed severe cyst adhesion to vital mediastinal structures and thus had incomplete resection. Multivariable logistic analysis revealed that a maximal cyst diameter greater than 5 cm was significantly associated with increased risks of operation time extension (OR = 2.106; 95% CI: 1.147–3.865, *P* = 0.016) and intraoperative blood loss increase (OR = 4.428; 95% CI: 1.243–16.489, *P* = 0.022). A total of 230 patients had follow‐up data. The median follow‐up time was 70 months (range, 36–210 months). No local recurrence was observed.

**Conclusions:**

Surgical resection by VATS may be recommended for PMC management as a primary therapeutic strategy. Cysts with a maximum diameter greater than 5 cm or cysts adjacent to vital mediastinal structures can increase the surgical difficulties.

**Key points:**

• **Significant findings of the study**

A diameter >5 cm and adhesions significantly increased the risk of operation time extension together with increased blood loss.

• **What this study adds**

Cysts with a diameter >5 cm or those adjacent to vital mediastinal structures increased the potential for surgical difficulties.

## Graphical abstract

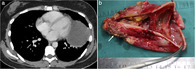

Surgical resection by VATS may be recommended for PMC management as a primary therapeutic strategy. The cysts with maximal diameter greater than 5 cm or the cysts that are adjacent to vital mediastinal structures can increase the surgical difficulties.

## Introduction

Primary mediastinal cysts (PMCs) are associated with developmental abnormalities of the primitive foregut or the precursors of the pericardium or pleura. They are rare benign masses, accounting for 12% to 18% of all mediastinal tumors, and contain a wide variety of pathologic types, such as bronchogenic cyst, thymic cyst, pericardial cyst, esophageal duplication cyst, and other miscellaneous types.^1^ PMCs are mainly revealed by the radiological procedures in routine medical examinations and are asymptomatic in most cases. Surgical resection of PMCs could confirm the pathological diagnosis, relieve the symptoms caused by the cysts, and prevent complications during conservative treatments.^2^, ^3^ However, guidelines for the surgical management of PMCs have not been established, and whether asymptomatic patients should be treated with surgery remains unclear. Video‐assisted thoracic surgery (VATS) has been commonly adopted to treat PMCs because of its minimally invasive nature. Here, we summarize our more than 15‐year experience to emphasize the advantages of performing VATS to treat PMCs, to investigate the clinical factors increasing surgical difficulties, and to provide diagnostic and surgical therapeutic recommendations. To the best of our knowledge, this is one of the largest single‐center study on experiences of performing VATS to treat PMCs.

## Methods

**Figure 1:**
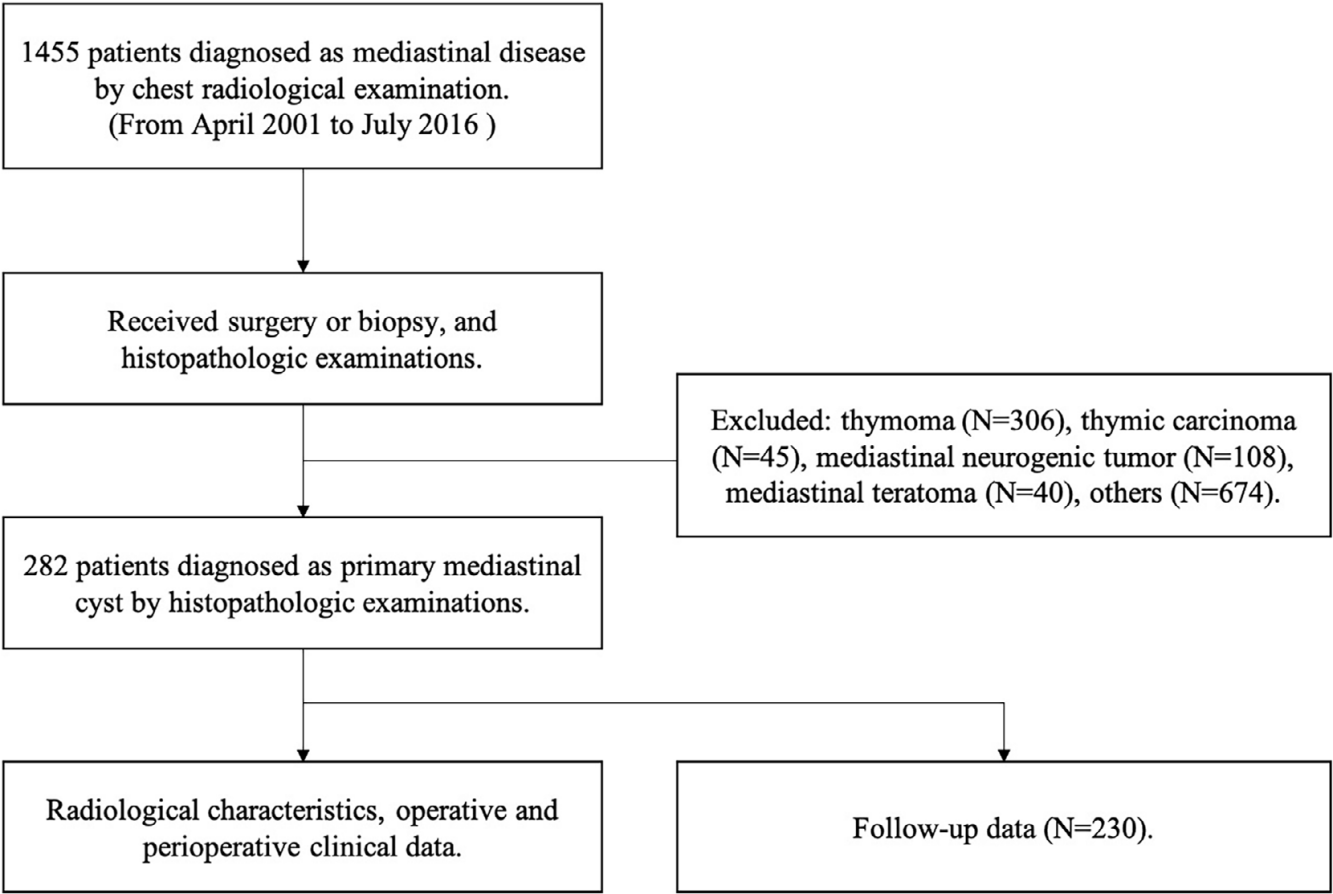
Flow diagram for the patient selection included in this study.

The medical records of 282 consecutive patients with a confirmed diagnosis of PMC who underwent surgical resection at Peking University People's Hospital from April 2001 to July 2016 were reviewed. The selection flow chart is shown in Fig 1. The criteria for surgical resection of PMC were: (i) patient with symptoms caused by a mediastinal cyst; (ii) the cyst grew progressively during the clinical observation; (iii) malignant tumor could not be ruled out by the preoperative radiological examination; and (iv) patients showed severe compression of the vital mediastinal structures (eg, pulmonary artery or main bronchus). All patients were evaluated by chest computed tomography (CT) prior to surgery and routine preoperative examinations which included complete blood test, echocardiography and pulmonary function test were also performed. The data of demographic characteristics, operative exploration, perioperative and follow‐up data were analyzed. Patients were followed‐up by telephone interview or outpatient clinic visit annually. The latest follow‐up was completed in July 2019.

Patients underwent general anesthesia, and VATS was performed on all the PMC patients. For patients with anterior mediastinal cysts, VATS was performed in the 30° lateral recumbent position, and the lateral recumbent position was used for patients with middle and posterior mediastinal cysts. Biportal or triportal VATS was performed.

Our surgical principles were: (i) lesions should be removed as completely as possible with a maximum effort to avoid cyst fluid leakage; (ii) if malignant tumor or thymoma could not be ruled out based on the preoperative imaging results and intraoperative examination for anterior mediastinal cysts, thymectomy should be performed; and (iii) because severe cyst adhesions to vital mediastinal structures might lead to incomplete resection, chemical agents or physical approach could be used to eliminate the residual cysts. At the end of surgery, a closed thoracic drainage tube was routinely inserted. Histopathologic examinations confirmed the diagnosis of mediastinal cysts, which included thymic, bronchogenic, pericardial, esophageal duplication, pleural, lymphangioma, and other types of mediastinal cysts.

## Statistical analysis

Data were analyzed using the Statistical Product and Service Solutions software, version 20.0 (SPSS, IBM Corporation, Armonk, NY, USA). Comparison of continuous variables was performed using a *t*‐test and analysis of variance (ANOVA). Chi‐square test was used to compare categorical variables. Univariate and multivariable logistic regression were used to identify independent predictors for long operation time (>120 minutes) and large blood loss (>100 mL). Predictors (*P* < 0.1) in the univariate analysis were incorporated into a multivariable analysis. *P*‐values < 0.05 were considered statistically significant. The study was approved by the Institutional Review Board of Peking University People's Hospital (IRB No. 2016PHB156‐01). Informed consent was obtained from all patients before surgery.

## Results

A total of 282 patients (159 women and 123 men) were included in the analyses. The mean age was 49.5 years (range, 4–75 years). Of the 282 patients, 78 (27.6%) were symptomatic. The most common symptom was cough (8.5%), followed by dyspnea (6.4%), chest pain (6.0%), dysphagia (1.4%), and fever (1.1%). Symptoms developed in 18 patients during a median 2.5‐year follow‐up time, and 15 (83.3%) of the 18 patients had cysts in the middle or posterior mediastinum. In addition, 50.7% of the PMC patients were correctly diagnosed by chest CT. The mean size of the mediastinal cysts was 4.2 ± 2.8 cm in the maximum diameter (range, 0.5–22.0 cm). The mean CT value was 25.7 ± 16.1 Hu (range: −8.0–67.0 Hu) on noncontrast CT. The CT value of bronchogenic cysts was higher than other types of mediastinal cysts (35.6 ± 14.7 vs. 19.0 ± 13.4 Hu, *P* < 0.001). According to the location of the cysts, we classified the patients into three groups: Group I (patients with anterior mediastinal cysts), Group II (patients with middle mediastinal cysts) and Group III (patients with posterior mediastinal cysts). The demographics and clinical characteristics of the three groups are shown in Table 1. The patients in Group III had the highest CT value (*F* = 5.356, *P* = 0.001). A significantly higher proportions of patients in Group II (13.3%) and III (17.9%) than in Group I (1.6%) developed symptoms during follow‐up (*F* = 7.304, *P* < 0.001).

**Table 1:** Demographic patient characteristics with primary mediastinal cysts divided into three groups according to location

Variables	Group I (*N* = 183)	Group II (*N* = 60)	Group III (*N* = 39)	*F*‐value	*P‐*value
Age (median, IQR)	55 (49–62)	41 (29–50)	41 (30–51)	48.201	<0.001
Sex (male/female)	74/109	30/30	19/20	0.974	0.406
Clinical symptom (%)	45 (24.6%)	24 (40.0%)	9 (23.1%)	0.466	0.706
Symptom developed during observation (%)	3 (1.6%)	8 (13.3%)	7 (17.9%)	7.304	<0.001
Cyst size (mean ± SD, range)	4.2 ± 3.0 (0.5–16.0)	4.4 ± 2.8 (0.5–22.0)	4.0 ± 1.6 (0.5–8.5)	0.253	0.859
Mean CT attenuation (mean ± SD, range)	23.8 ± 14.2 (0–52.0)	24.1 ± 16.3 (−8.0–60.0)	35.5 ± 20.1 (0–67.0)	5.356	0.001
Diagnosed as mediastinal cyst by CT (%)	98 (53.6%)	27 (45.0%)	18 (46.2%)	0.885	0.449
Pathology				4.730	0.003
Thymic cyst	118	1	1		
Bronchogenic cyst	48	41	31		
Pericardial cyst	13	12	1		
Esophageal cyst	1	3	3		
Pleural cyst	2	1	0		
Lymphangioma cyst	0	1	0		
Others	1	1	3		

Group I: Patients with anterior mediastinal cyst. Group II: Patients with middle mediastinal cyst. Group III: Patients with posterior mediastinal cyst. IQR, interquartile range; SD, standard deviation

Complete cyst resection by VATS was achieved in 278 patients. Three patients were converted to thoracotomy because of severe cyst adhesions to the mediastinal structure or a giant cyst in the lower esophagus. The mean operation time was 102.4 ± 40.9 minutes (range 25–360 minutes), and the mean intraoperative bleeding was 52.4 ± 75.1 mL. The median duration of thoracic drainage was three days (range, 1–20 days), and the median hospital stays was four days (range 2–23 days). The incidence rate of overall postoperative complication was 5.7%. There was no significant difference regarding the above data among the three groups. However, incomplete cyst resection was seen in seven patients with bronchogenic cysts because of severe cyst adhesion to tracheal wall membrane and pulmonary artery wall, and all the cysts were in the middle mediastinum (Table 2). Of the 282 patients, 230 (81.5%) had follow‐up data. The follow‐up duration was between 36 months and 210 months with a median of 70 months. No late complication or recurrence was observed in the 230 patients.

**Table 2:** Peri‐ and postoperative results of patients with primary mediastinal cysts

Variables	Group I (*N* = 183)	Group II (*N* = 60)	Group III (*N* = 39)	*F*‐value	*P‐*value
Operation time (mean ± SD, range)	102.5 ± 37.6 (40.0–250.0)	103.3 ± 37.8 (25.0–190.0)	100.5 ± 58.4 (40.0–360.0)	0.055	0.947
Blood loss (mean ± SD, range)	49.5 ± 58.9 (5.0–500.0)	57.2 ± 71.8 (10.0–450.0)	58.3 ± 130.3 (5.0–600.0)	0.376	0.687
Adhesions around the cyst (%)	53 (29.0%)	21 (35.0%)	14 (35.9%)	0.612	0.543
Surgery‐related complications (%)	2 (1.1%)	4 (6.7%)	2 (5.1%)	1.355	0.260
Esophageal injury	0	2	2		
Tracheal injury	0	2	0		
Pericardial injury	2	0	0		
Incomplete excision of the cyst wall (%)	0	7 (11.7%)	0	14.504	<0.001
Chest tube duration (median, IQR)	3 (2–3)	2 (2–3)	2 (1–3)	1.866	0.157
Duration of hospital stay (median, IQR)	4 (4–6)	4 (3–5)	4 (3–5)	1.907	0.150
Postoperative complications (%)	9 (4.9%)	4 (6.7%)	3 (7.7%)	0.299	0.742
Arrhythmia	5	1	1		
Pneumonia	2	1	1		
Prolonged air leakage (≥7 days)	0	1	0		
Chylothorax	2	1	1		
Surgical approaches by VATS				17.615	<0.001
Biportal	18	14	18		
Triportal	164	45	19		
Conversion of surgical approach	1	1	2	1.916	0.149
Recurrence/follow‐up patients	0/159	0/42	0/29		

Group I: Patients with anterior mediastinal cyst. Group II: Patients with middle mediastinal cyst. Group III: Patients with posterior mediastinal cyst. IQR, interquartile range; SD, standard deviation.

**Figure 2:**
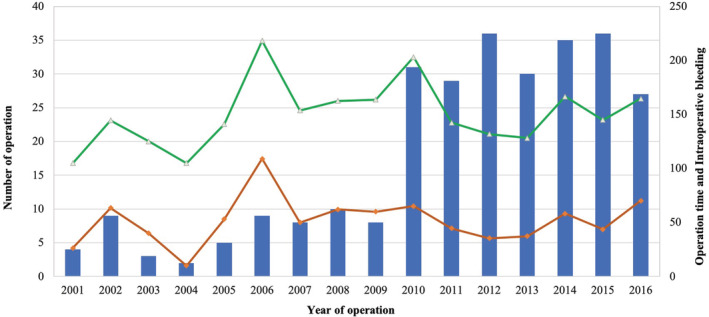
The number of operations, operative duration and intraoperative bleeding of each year (2001–2016). (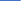) Number of operation, (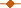) Intraoperative bleeding (mL), and (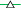) Operation time (minutes).

The number of operations, operative duration and intraoperative bleeding of each year are illustrated in Fig 2. There were no differences in terms of intraoperative bleeding (64.4 vs. 50.8 mL, *P =* 0.337), operation time (91.1 vs.103.9 minutes, *P =* 0.097) and postoperative complications (12.5% vs. 4.8%, *P* = 0.076) between the early phase group (2001–2006) and late phase group (2007–2016). To identify the factors that could increase operative duration and intraoperative bleeding, univariate and multivariable logistic regression analysis were performed. The multivariable analysis revealed that a maximal diameter greater than 5 cm (OR = 2.106; 95% CI: 1.147–3.865, *P* = 0.016), mediastinal structure damage (OR = 10.875; 95% CI: 2.015–58.681, *P* = 0.006), and cyst adhesion to the mediastinal structure (OR = 2.345; 95% CI: 1.303–4.223, *P* = 0.005) were significantly associated with longer operation time (Table 3). Furthermore, the multivariable analysis demonstrated that a maximal diameter greater than 5 cm (OR = 4.428; 95% CI: 1.243–16.489, *P* = 0.022) and cyst adhesion to the mediastinum (OR = 9.617; 95% CI: 2.003–46.169, *P* = 0.005) were significantly associated with a risk of increased blood loss (Table 4).

**Table 3:** Univariate and multivariable logistic regression analysis for factors extending operative duration†

	Univariate analysis			Multivariable analysis		
Variables	OR	95% CI	*P*‐value	OR	95% CI	*P*‐value
Age (≤50 vs. >50 years)	0.777	0.449–1.347	0.369	—	—	—
Sex (female vs. male)	1.246	0.718–2.160	0.433	—	—	—
Clinical symptom (yes vs. no)	1.431	0.783–2.616	0.243	—	—	—
Location (Group I vs. Group II and III)	1.234	0.700–2.174	0.467	—	—	—
Preoperative follow‐up time (≤2 vs. >2 years)	1.579	0.649–3.843	0.311	—	—	—
Maximal diameter (>5 vs. ≤5 cm)	1.998	1.111–3.558	0.019	2.106	1.147–3.865	0.016
Diagnosed as mediastinal cyst by CT (yes vs. no)	0.792	0.457–1.372	0.405	—	—	—
Mediastinal structure damage (yes vs. no)	10.475	2.062–53.221	0.001	10.875	2.015–58.681	0.006
Adhesions to mediastinal structure (yes vs. no)	2.378	1.353–4.221	0.002	2.345	1.303–4.223	0.005

† The operation time prolonged is defined as the operation time was more than 120 minutes.

**Table 4:** Univariate and multivariable logistic regression analysis for factors that increased the blood loss†

	Univariate analysis			Multivariable analysis		
Variables	OR	95% CI	*P*‐value	OR	95% CI	*P*‐value
Age (≤50 vs. >50 years)	0.346	0.090–1.331	0.107	—	—	—
Sex (female vs. male)	0.730	0.209–2.552	0.621	—	—	—
Clinical symptoms (yes vs. no)	3.317	0.982–11.201	0.042	3.612	0.876–14.897	0.076
Location (Group I vs. Group II and III)	1.569	0.467–5.277	0.463	—	—	—
Preoperative follow‐up time (≤2 vs. >2 years)	0.734	0.212–2.601	0.767	—	—	—
Maximal diameter (>5 vs. ≤5 cm)	5.436	1.543–19.151	0.004	4.428	1.243–16.489	0.022
Diagnosed as mediastinal cyst by CT (yes vs. no)	1.174	0.350–3.938	0.795	—	—	—
Mediastinal structure damage (yes vs. no)	0.960	0.937–0.983	0.563	—	—	—
Adhesions to mediastinal structure (yes vs. no)	10.937	2.311–51.756	<0.001	9.617	2.003–46.169	0.005

† The blood loss increased is defined as the blood loss is more than 100 mL.

## Discussion

PMCs are rare congenital benign lesions, which originate from various embryological tissue types. However, they have similar clinical manifestations and imaging features. The two most common histological variants of PMCs are bronchogenic and thymic cysts, accounting for 85% in our series. Approximately 64%–80% of patients with PMCs were asymptomatic. The symptoms of PMCs are mainly caused by compression or stimulation of the respiratory tract, lung or esophagus.^1^, ^2^ Moreover, some studies have indicated that about 45%–58% of asymptomatic PMC patients ultimately develop symptoms, and the cyst could grow or rupture, leading to a worsening of symptoms. Malignancy development in PMCs has been reported but is very rare.^1^, ^2^, ^3^ The optimal therapeutic management of PMC is still unknown because of the unpredictability of PMC clinical behavior. Therefore, we reviewed our experience in the clinical management of 282 patients with PMC to provide reasonable diagnostic and surgical therapeutic recommendations.

The preoperative diagnosis of a PMC mainly depends on imaging examinations. Chest CT is commonly used to identify the size and shape of cysts and reveal the relationships between PMCs and their surrounding vital mediastinal structures. The characteristic CT presentations of PMCs include: (i) in the mediastinum; (ii) manifest as fluid attenuation; (iii) a round‐like shape with a thin wall, well‐circumscribed, homogeneous attenuation; and (iv) weak or no enhancement on contrast‐enhanced CT.^4^, ^5^ However, only 50.7% of the patients in our series showed these characteristic CT presentations. Previous studies have showed that about 50% of PMC patients demonstrated soft‐tissue attenuation on chest CT. Therefore, it is challenging to distinguish mediastinal cysts from solid neoplasm because cysts usually show high attenuation on chest CT, which might be caused by protein‐rich cyst fluid.^5^, ^6^, ^7^ McAdams *et al*.^7^ reported a series of 20 patients with bronchogenic cysts who underwent both chest CT and chest MRI, and they found that in nine patients these demonstrated a fluid‐like signal on MRI but were present as soft‐tissue‐like on CT scan. In the study by Tomiyama *et al*.^8^ the rates of accurate diagnosis based on CT and MRI for thymic cysts were 46.0% and 71.0% (*P* < 0.05), respectively, suggesting that MRI might reveal more diagnostic information than CT. Therefore, MRI is an important supplementary method for the diagnosis of mediastinal cysts.

Most surgeons agree that symptomatic patients, or patients with progressively growing cysts should be treated with surgical resection. However, how to manage asymptomatic patients remains controversial.^3^, ^9^ PMCs are congenital benign disorders, and they rarely lead to serious clinical symptoms or malignant transformation. Most PMCs are slow‐growing and are easily diagnosed by advanced imaging examinations (eg, contrast‐enhanced CT or MRI). In our study, only one patient with a thymic cyst had a simultaneous type B1 thymoma. In the review by Kirmani and Sogliani,^3^ data of 683 PMC patients from 23 papers were analyzed. They found that the longest observation duration for asymptomatic patients was 22 years and only 0.7% patients developed malignant transformation in the cysts. Hence, for asymptomatic PMC patients, if their imaging examination can reveal characteristic mediastinal cyst presentations, cysts that can be easily distinguished from solid or malignant tumors, cysts with a relatively small size, or cysts located away from the mediastinal vital structure, conservative management could be an alternative therapeutic strategy. Nevertheless, these patients should be followed‐up routinely with imaging examinations.

**Figure 3:**
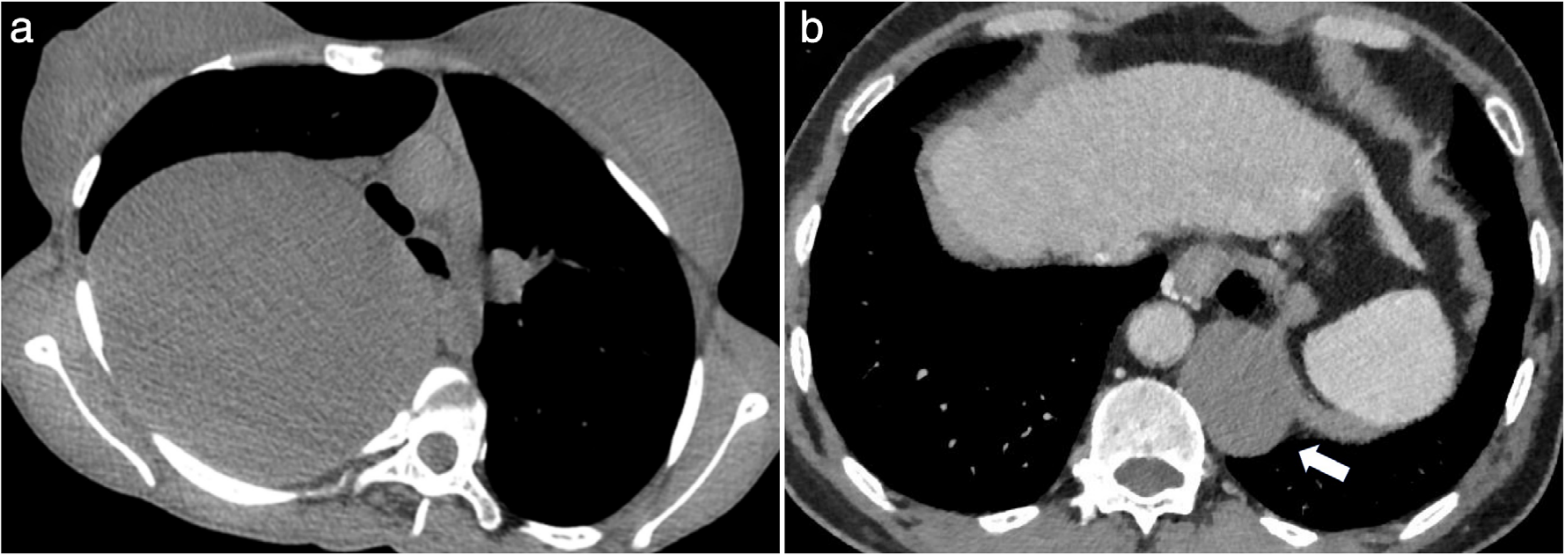
Chest CT showed two primary mediastinal cysts with serious complications caused by the compression of the cysts. (**a**) A giant mediastinal bronchogenic cyst compressed the right thoracic cavity severely and led to the deformity of the trachea. (**b**) Compression of the esophagus by an esophageal cyst which led to dysphagia (arrow).

**Figure 4:**
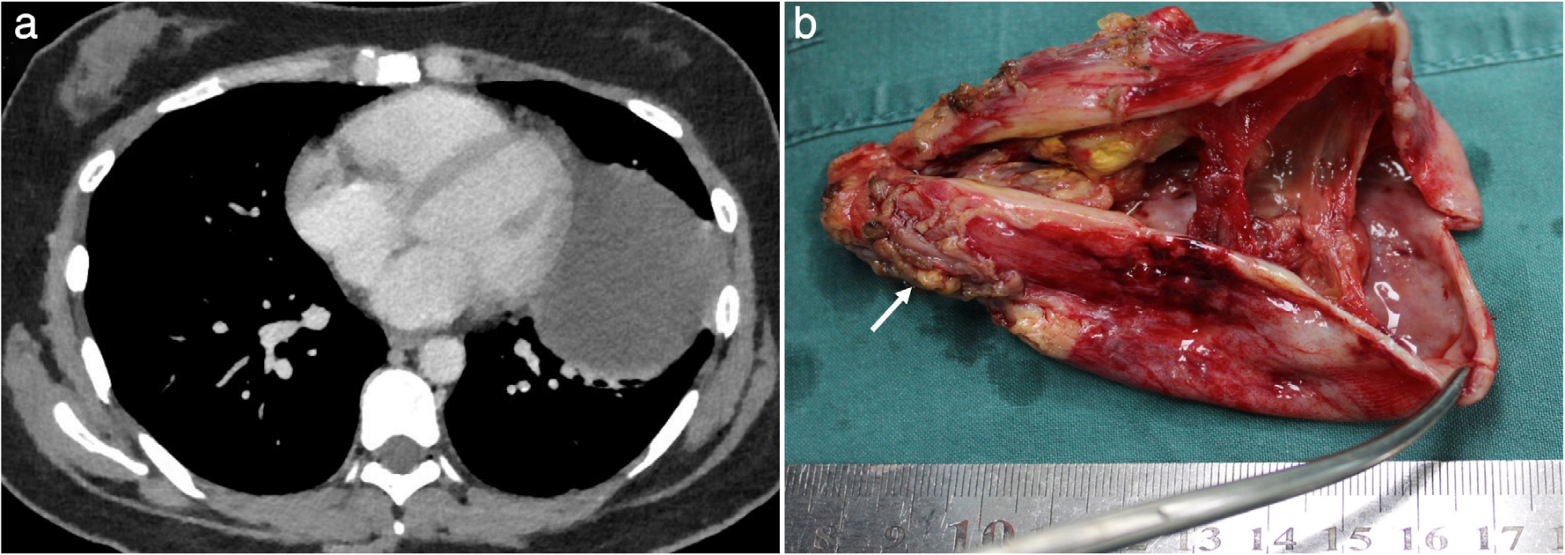
Chest CT and the resected specimen performance of pericardial cysts with severe adhesion to the left lung and chest wall which caused a severe inflammatory reaction around the cyst (arrow).

Previous studies have demonstrated that about 45% of asymptomatic patients might ultimately develop symptoms during follow‐up.^3^ Cyst rupture can lead to a pleural effusion, infection or hemorrhage in the thoracic cavity, and some serious complications caused by the compression of cyst (Fig 3), such as superior vena cava obstruction, dyspnea, and arrhythmia, could develop occasionally in some cases.^3^, ^9^, ^10^ The possibility of malignant transformation cannot be ruled out, especially for the cases with atypical imaging performance.^3^, ^10^ PMCs can grow continuously without inducing any symptom or complication. In addition, the growth of cysts may cause severe cyst adhesion to the adjacent mediastinal structures or stimulate a serious inflammation (Fig 4). These conditions make surgical treatment for PMC increasingly challenging and increase the risk of perioperative complications.^11^ In our series, 18 PMC patients developed symptoms eventually during the follow‐up, and 83.3% had middle or posterior mediastinal cysts, all adjacent to the tracheobronchial tree. Therefore, surgical resection of PMCs may be recommended to patients who could potentially develop symptoms or clinical complications in the future.

An aggressively growing cyst could result in severe adhesion to the mediastinal structures, which makes complete surgical resection of the cyst very challenging.^12^ Fievet *et al*.^9^ investigated the outcomes of 36 patients with mediastinal bronchogenic cysts and found that surgical resection of early‐stage cysts can reduce inflammatory lesion development and decrease the incidence rate of perioperative complications. In our series, the multivariable logistic regression analysis revealed that cysts with a maximal diameter greater than 5 cm increased the risk of operative duration extension by 2.106 times and the risk of blood loss increase by 4.428 times. Hence, the process of cyst growth might increase the surgical difficulties. In our center, the following criteria is used to determine whether a patient needs surgical resection of PMC: (i) patients with symptoms caused by a mediastinal cyst; (ii) the cyst grew progressively during clinical observation; (iii) malignant tumor could not be ruled out by preoperative imaging examination; and (iv) patients showed severe compression of the vital mediastinal structures (eg, pulmonary artery or main bronchus).

Mouroux *et al*.^13^ reported the first cases where VATS was performed to treat mediastinal cysts in 1991. Since that time, VATS has been increasingly adopted to treat various mediastinal cysts.^2^, ^12^, ^13^, ^14^, ^15^ Guo and colleagues^16^ compared VATS versus traditional thoracotomy in patients undergoing surgical resection of PMCs and confirmed that VATS could reduce the operative duration and intraoperative blood loss without increasing the risks of incomplete resection and intraoperative and postoperative complications. In our series, VATS was initially performed for all patients. Only four patients (1.4%) were converted to thoracotomy because of severe cyst adhesions or the difficulties of cyst access. Therefore, VATS could be a primary therapeutic option to treat PMCs.

Total cystectomy involving resection of the entire cyst wall is considered the standard surgical treatment for PMCs. However, there are several clinical problems that should be taken into consideration in clinical practice: (i) for anterior mediastinal cysts, whenever thymoma, cystic degradation of thymoma or other malignant tumors in the thymus cannot be excluded based on preoperative imaging results and intraoperative examination, thymectomy is recommended for the purpose of an accurate diagnosis and a complete resection; and (ii) for middle mediastinal cysts, whenever a severe cyst adhesion to the vital mediastinal structures (eg, the membranous wall of trachea, the esophageal mucosa or the great vessels) is present, complete excision of the PMCs without vascular or tracheal damages may be very challenging. We recommend using physical approaches (eg, electrocautery) or chemical agents (eg, alcohol or iodine tincture) to destroy the residual cyst wall when there has been an incomplete cyst resection.

This study was a single‐center and retrospective analysis. The sample size was relatively small, and follow‐up duration also relatively short. Multicenter studies with higher volumes of PMC patients are necessary to confirm the conclusions of the study.

In conclusion, surgical resection of early‐stage PMC by VATS is recommended as a primary therapeutic option for clinical management of PMCs. Cysts with a maximal diameter greater than 5 cm or cysts that are adjacent to the bronchial tree, esophagus or the pulmonary vessels can increase surgical difficulties.

## Disclosure

The authors have no conflicts of interest to declare.
